# Availability and distribution of human resources for provision of comprehensive emergency obstetric and newborn care in Afghanistan: a cross-sectional study

**DOI:** 10.1186/s13031-015-0037-6

**Published:** 2015-03-16

**Authors:** Manizha Faqir, Partamin Zainullah, Hannah Tappis, Jaime Mungia, Sheena Currie, Young Mi Kim

**Affiliations:** International Rescue Committee, 3 Bloomsbury Place, London, WC1A 2QL UK; Jhpiego/Afghanistan, an affiliate of Johns Hopkins University, Shar-e-Naw, Street 3, House 265, Kabul, Afghanistan; Jhpiego/USA, an affiliate of Johns Hopkins University, 1615 Thames St., Baltimore, MD USA

**Keywords:** Human resources, Emergency obstetric care, Maternal health, Neonatal health, Health services, Afghanistan

## Abstract

**Background:**

For over a decade, Afghanistan’s Ministry of Public Health and its international development partners have invested in strengthening the national health workforce and establishing a system of primary health care facilities and hospitals to reduce the high levels of maternal and child mortality that were documented shortly after the fall of the Taliban in 2001. Significant progress has been made, but many challenges remain. The objective of this study is to assess the availability and distribution of human resources for round-the-clock comprehensive emergency obstetric and newborn care service provision in secure areas of Afghanistan in order to inform policy and program planning.

**Methods:**

A cross-sectional assessment was conducted from December 2009 to February 2010 at the 78 accessible facilities designated to provide emergency obstetric and newborn care in Afghanistan. The availability of staff on call 24 hours a day, seven days a week; involvement of staff in essential clinical functions; turnover rates; and vacancies were documented at each facility. Descriptive statistics were used to summarize results.

**Results:**

All facilities assessed had at least one midwife on staff, but most did not meet the minimum staffing requirements set in national guidelines. Given that all facilities assessed are considered referral centers for lower-level clinics, the lack of doctors at 5% of facilities, lack of anesthetists at 10% of facilities and lack of obstetrician/gynecologists at 51% of facilities raises serious concerns about the capacity of the health system to respond with lifesaving care for women with obstetric complications.

**Conclusions:**

While the government continues its efforts to increase the number of qualified female health professionals in Afghanistan after decades with little female education, innovative strategies are needed to facilitate deployment, skill-development and retention of female healthcare providers in underserved areas.

## Background

The World Health Organization estimates that at least 88–98% of maternal deaths can be averted with timely access to lifesaving care [1]. In many countries, progress towards Millennium Development Goal 5—reducing maternal mortality—and the provision of universal access to reproductive health are constrained by supply-side limitations, including shortages of skilled health workers needed to deliver evidence-based interventions [2-4]. Availability of competent human resources for health continues to be a challenge in many countries and is often highlighted as a priority at high-level global discussions and advocacy events [5].

In Afghanistan, where the maternal mortality ratio is currently estimated at 327 per 100,000 live births, social development efforts are hindered by financial dependence on international donors, widespread poverty, illiteracy, and ongoing insecurity in many parts of the country [6]. Socio-cultural norms dictate that maternal health services be provided by female clinicians [7]. One of Afghanistan’s major challenges is the availability of human resources for health, which is attributed to out-migration of skilled professionals, maldistribution of staff, especially of doctors with an urban bias, and ongoing restrictions on girls’ and women’s education. In the recently published State of the Worlds Midwifery Report, the met need for reproductive, maternal, newborn, and child health services in Afghanistan was only 23% and there were only 400 staff classified as obstetricians/gynecologists [8]. Even with support from international donors to establish pre-service education programs to increase the midwifery workforce and in-service clinical refresher training programs for physicians and midwives, gaps in health service coverage remain. Current statistics indicate the number of certified midwives in Afghanistan has increased from 467 in 2003 to 3,807 in 2013 [9]. However, this is still less than half of the projected number of midwives needed to serve the population, which is calculated as one midwife for every 175 births with a 15% attrition rate that is in line with international guidelines [10].

The primary strategy for scaling up access to maternal health services in Afghanistan has been implementation of a Basic Package of Health Services (BPHS) at primary health care facilities (i.e. health posts, basic health centers, comprehensive health centers, and district hospitals) and an Essential Package of Hospital Services (EPHS) at provincial hospitals, regional hospitals, and specialized hospitals [11,12]. According to BPHS and EPHS guidelines, all hospitals are expected to provide round-the-clock emergency obstetric and newborn care (EmONC) services, particularly the nine signal functions that directly treat the obstetric and newborn complications that are the proximate causes of maternal and neonatal death. The seven signal functions characterizing basic EmONC (BEmONC) are: administering parenteral antibiotics, administering parenteral anticonvulsants, administering parenteral uterotonics, manual removal of the placenta, removal of retained products of conception using manual vacuum aspiration, assisted vaginal delivery with a vacuum extractor or forceps, and newborn resuscitation with a bag and mask. The two additional signal functions that comprise comprehensive EmONC (CEmONC) are blood transfusion and cesarean surgery. Comprehensive health centers are generally expected to provide only BEmONC services. However, in remote districts where access to a hospital is limited, some comprehensive health centers have been upgraded to small hospitals that can perform CEmONC services. Provision of all nine functions requires a team of skilled personnel, including obstetricians/gynecologists or surgeons (to provide caesarean surgery), midwives, nurses, and anesthetists (broadly defined to include medical doctors and nurses with specialized training in anesthesia) to support surgical care and blood transfusion services. Staffing requirements are summarized by facility type in Table 1; the capacity of the health workforce to perform selected EmONC signal functions is reported elsewhere [13].

**Table 1:** **BPHS/EPHS guidelines for BEmONC and CEmONC provision and staffing, by facility type** [10]

**Facility type**	**Catchment population**	**Services**		**Cadre**									**Notes**
		**BEmONC**	**CEmONC**	**Anesthetist**	**Lab technician**	**Medical doctor**	**Midwife**	**Nurse**	**Obstetrician/gynecologist**	**Pediatrician**	**Pharmacist**	**General Surgeon**	
Health Post	1,000–1,500	No	No	0	0	0	0	0	0	0	0	0	Operated by volunteer male and female community health workers
Basic Health Center	15,000–30,000	Yes	No	0	0–1	1	1	1	0	0	0	0	Preference is for a female doctor and nurse, but this is not a requirement.
Comprehensive Health Center	30,000–60,000	Yes	No	0	1	2	2	2	0	0	1	0	One doctor and one nurse should be female. For upgraded facilities (“CHC plus”), one obstetrician/gynecologist, one surgeon, and one anesthetist are also required.
District Hospital	100,000–300,000 (25–75 beds)	Yes	Yes	1	2	4	4	12	1	1	1	2	At least 50% of doctors and nurses should be female. Two of the nurses are to be dedicated for anesthesia services. More advanced technicians are required for blood bank, x-rays, etc.
Provincial Hospital	(75–250 beds)	Yes	Yes	2	2	10	8	40	3	2	2	4	At least 50% of doctors and nurses should be female. Four of the nurses are to be dedicated for anesthesia services. More advanced technicians are required for blood bank, x-rays, etc.
Regional Hospital	(300–450 beds)	Yes	Yes	3–4	4	20	12	40	5	4	3	6	At least 50% of doctors and nurses should be female. Four of the nurses are to be dedicated for anesthesia services. More advanced technicians are required for blood bank, x-rays, etc.
Specialized Hospital	Not specified	Yes	Yes	NA									Staff numbers vary according to need.

With limited human resources for health service provision, careful planning and management of the health workforce, including recruitment, selection, education, deployment, and supervision of workers, is critical for advancing access to maternal and newborn health services in Afghanistan. The objective of this study is to assess the availability and distribution of human resources for round-the-clock CEmONC service provision in secure areas of Afghanistan. Mapping current gaps and needs will help inform continued health workforce strengthening efforts in Afghanistan.

## Methods

A cross-sectional assessment of the availability and utilization of CEmONC services in Afghanistan was conducted from December 2009 to February 2010. It examined all public health facilities designated to provide CEmONC services, including district hospitals, provincial hospitals, regional hospitals, and national specialized hospitals, as well as comprehensive health centers upgraded to provide CEmONC services in districts without a referral hospital.

At the time of the assessment, 127 health facilities provide CEmONC services across Afghanistan’s 34 provinces. Of these, 49 facilities were inaccessible due to security constraints at the time of the assessment. Therefore, the assessment was limited to 78 facilities located in comparatively secure areas of the country. They included 9 comprehensive health centers, 34 district hospitals, 25 provincial hospitals, 5 regional hospitals, and 5 specialized hospitals. The assessment team consisted of 6 doctors and 38 midwives. All were experienced service providers and had helped collect data for previous studies in Afghanistan. The assessors attended one week of training on the assessment protocol, data collection tools, and research ethics. After the training, the assessment teams pre-tested tools at health facilities in Kabul City to ensure intra- and inter-assessor reliability. They visited each facility for one to three days to collect data. Health facilities were not informed in advance about the assessors’ visit. Upon arrival at a facility, assessors obtained consent from the facility’s medical director and held an introductory meeting with key informants, including staff in charge of maternity, surgery, pharmacy, and laboratory departments.

To investigate the availability of human resources for CEmONC provision at each facility, assessors used modified tools based on the Averting Maternal Death and Disability Program’s Needs Assessment Toolkit [14]. At each facility, payroll and attendance records were reviewed to determine the number and cadre of staff hired and dismissed/resigned during the past 12 months, as well as those actively employed at the time of the assessment. The head of the maternity ward or facility was then interviewed to determine which staff provided CEmONC signal functions and other essential services. In the event a cadre was reported to provide services not typically in the job description for that cadre, data collectors requested to see clinical records to verify that cadre’s involvement. If records confirmed involvement of the cadre, this was recorded in the data collection tool. If involvement could not be verified, data collectors did not record that cadre as performing the signal function. As CEmONC services should be provided 24 hours a day, seven days a week, the head of the maternity ward or facility was also asked about the availability of various staff on duty (physically present at the facility) and on call (contactable but not physically present at the facility) during weekdays, weekends, day shift, and night shift; responses were cross-checked against facility attendance records.

Turnover rates were calculated for each facility as the number of positions of a given cadre vacated during 2009, divided by the average number employed that year. This number includes all health workers leaving their post in 2009, whether due to transfers, dismissal, retirement, or other reasons [15]. Average turnover rates were then calculated as the simple average of the cadre-specific turnover rates across all facilities of that type. Minimum, maximum, and average turnover rates for each cadre and facility-type were examined.

The availability of staff reported to provide CEmONC services was analyzed by signal function; facility type; facility location in a district with >50%, ≤ 50%, or none of the population residing in urban areas; and region of the country. Chi square tests and linear regression models were used to identify factors associated with the availability of staff required by their job description to perform CEmONC services. Analyses were conducted using STATA version 11 with a type 1 error of 0.05 [16]. The assessment was approved by the institutional review boards of the Afghanistan Public Health Institute (IRB #2333) and the Johns Hopkins Bloomberg School of Public Health (IRB # 2359).

## Results

### Availability of human resources to provide CEmONC functions

**Figure 1:**
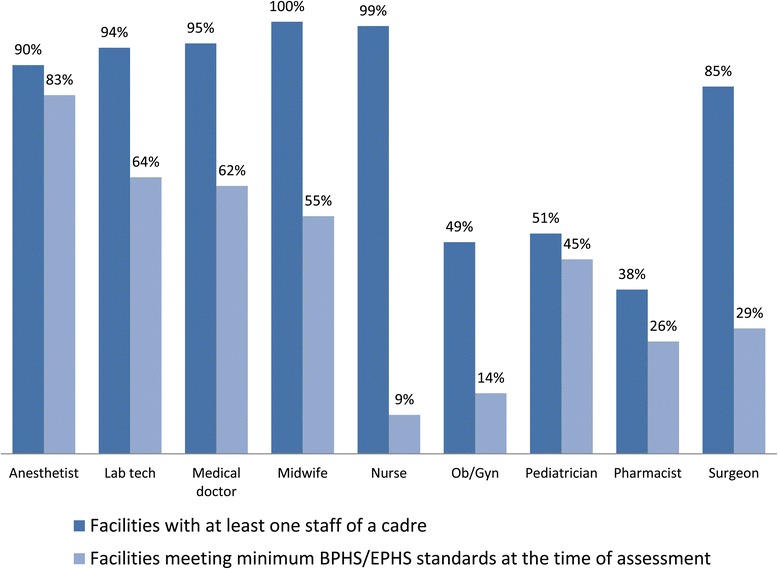
**Percentage of designated CEmONC facilities with at least one staff of a cadre, and percentage meeting minimum BPHS/EPHS staffing requirements at the time of assessment.**

Figure 1 shows the percentage of health facilities with at least one of a cadre on staff and the percentage meeting minimum staffing requirements for CEmONC provision, by facility type.

All facilities assessed had at least one midwife on staff, but most did not meet the minimum staffing requirements set out in the BPHS and EPHS guidelines. While 95% of facilities assessed had a medical doctor and 90% had an anesthetist on staff, only 49% had an obstetrician/gynecologist. A much smaller percentage met the minimum BPHS/EPHS staffing requirements for each position; only 55% (43) had the required number of midwives, 14% (11) had the required number of obstetrician/gynecologists, and only 9% (7) had the required number of nurses.

Staff turnover was high and a challenge for many facilities. Table 2 presents the average turnover rate for each cadre of staff, by facility type. At hospitals surveyed, two-fifths of doctors and laboratory technicians left their posts during the 12 months preceding the assessment. Comprehensive health centers experienced considerably higher turnover than hospitals: every obstetrician/gynecologist, general surgeon, and laboratory technician assigned to these facilities left over the 12 month period preceding the assessment, as did two-fifths of medical doctors and anesthetists. The average turnover rate ranged from 0% for medical doctors at regional hospitals and pharmacists at regional and specialty hospitals, to 125% for obstetrician/gynecologists at comprehensive health centers (turnover rates at the two comprehensive health centers reporting obstetrician/gynecologists on staff in 2009 were 50%^a^ and 200%^b^).

**Table 2:** Minimum, maximum and averageturnover rate for available cadres, by facility type *

		**Anesthetist**	**Labtechnician**	**Medical doctor**	**Midwife**	**Nurse**	**Obstetrician/gynecologist**	**Pediatrician**	**Pharmacist**	**General surgeon**
**Comprehensive Health Center (n = 9)**	# of facilities with cadre	5	7	8	9	9	2	0	0	9
	Min-max turnover rate	N/A (0%)	0–50%	0-100%	0-100%	0-100%	0-200%	0	0	0-200%
	***Average turnover rate***	***0%***	***7%***	***40%***	***24%***	***18%***	***125%***	N/A	N/A	***92%***
**District Hospital (n = 34)**	# of facilities with cadre	33	32	34	34	34	31	32	12	34
	Min-max turnover rate	0-200%	0-100%	0-80%	0-100%	0-67%	0-200%	0-200%	0-100%	0-200%
	***Average turnover rate***	***19%***	***11%***	***13%***	***19%***	***12%***	***42%***	***30%***	***12%***	***27%***
**Provincial Hospital (n = 25)**	# of facilities with cadre	25	25	25	25	25	25	25	12	25
	Min-max turnover rate	0–100%	0–150%	0–72%	0–67%	0–67%	0–100%	0–300%	0–50%	0–200%
	***Average turnover rate***	***19%***	***15%***	***15%***	***11%***	***12%***	***17%***	***18%***	***10%***	***27%***
**Regional Hospital (n = 5)**	# of facilities with cadre	5	5	5	5	5	5	5	3	5
	Min–max turnover rate	0–18%	0–143%	N/A (0%)	0–24%	0–83%	0–50%	0–20%	N/A (0%)	N/A (0%)
	***Average turnover rate***	***5%***	***29%***	***0%***	***8%***	***20%***	***10%***	***4%***	***0%***	***0%***
**Specialized Hospital (n = 5)**	# of facilities with cadre	5	5	5	5	5	5	5	3	5
	Min–max turnover rate	0–15%	0–60%	0–4%	0–40%	0–53%	0–25%	0–25%	0%	0–12%
	***Average turnover rate***	***5%***	***12%***	***1%***	***12%***	***20%***	***13%***	***5%***	***0%***	***2%***
**All designated CEmONC facilities (n = 78)**	# of facilities with cadre	71	73	74	78	77	41	41	30	69
	Min–max turnover rate	0–200%	0–150%	0–100%	0–100%	0–100%	0–200%	0–300%	0–100%	0–200%
	***Average turnover rate***	***16%***	***13%***	***15%***	***16%***	***14%***	***28%***	***18%***	***9%***	***27%***

^*^ *Minimum and maximum cadre-specific turnover rates are the lowest and highest facility-level turnover rates for each facility type. Average turnover rates are the arithmetic mean of cadre-specific turnover rates across all facilities with a given cadre employed at any point during 2009.*

Table 3 shows the percentage of facilities with essential staff available onsite during weekday and weekend day and night shifts, or on call. At the time of the study, Fridays were considered weekends (in Kabul, Thursdays were considered the weekend) and Saturdays through Thursdays were considered workweeks. Availability of cadres for day and night shifts during the week and over the weekend was most consistent for midwives, nurses, and medical doctors. Presence of a midwife and doctor onsite ranged from 97.4% for midwives and 88.3%for doctors on weekends nights and100% and 94.8%on daytime weekday shifts, respectively. Obstetrician/gynecologists, pediatricians, and surgeons were more likely to be on call than onsite, and in many cases were unavailable at various days and times. Presence of an obstetrician/gynecologist on site ranged from 47.2% of facilities during the daytime work week to 12.5% of facilities on weekend nights; 36.1%of facilities had this essential cadre on call.

**Table 3:** **Percent of designated CEmONC facilities with essential staff on site and on call at health facilities at the time of the assessment**

**Cadre**	**Number of facilities with cadre employed**	**Proportion of facilities with the cadres reporting their availability at various working times**				
		**Daytime weekday**	**Night weekday**	**Daytime weekend**	**Night weekend**	**On call**
Anesthetist	70	85.3%	53.3%	46.7%	46.7%	44.0%
Lab tech	73	90.5%	73.0%	74.3%	68.9%	24.3%
Medical doctor	74	94.8%	92.2%	89.6%	88.3%	7.8%
Midwife	78	100.0%	98.7%	97.4%	97.4%	1.3%
Nurse	77	97.4%	97.4%	97.4%	97.4%	0.0%
Obstetrician/gynecologist	38	47.2%	15.3%	18.1%	12.5%	36.1%
Pediatrician	40	46.6%	20.5%	19.2%	20.5%	35.6%
Pharmacist	30	96.7%	60.0%	63.3%	50.0%	10.0%
General surgeon	66	74.4%	42.3%	35.9%	38.5%	47.4%

Table 4 presents provision of signal functions by various cadres. Overall,49% of facilities performed all CEmONC functions during the three months preceding the assessment, while 61% performed a subset (seven of the nine CEmONC services) that comprise BEmONC. At hospitals, obstetrician/gynecologists, medical doctors, and midwives performed most of the signal functions. Facilities reported involvement of medical doctors, obstetrician/gynecologists, midwives, general surgeons, pediatricians, nurses, anesthetists, and lab technicians in administration of parenteral antibiotics, blood transfusions, and cesarean deliveries. Other signal functions were provided predominantly by midwives and medical doctors, or specialists where available.

**Table 4:** **Facilities performing CEmONC functions during three months prior to assessment, with reported involvement by cadre**

**Signal function**	Number of facilities reporting performing function*	**% of facilities reporting involvement of each cadre in provision of signal function**							
		**Anesthetist**	**Lab technician**	**Medical doctor**	**Midwife**	**Nurse**	**Obstetrician/gynecologist**	**Pediatrician**	**General surgeon**
**BEmONC functions**									
Parenteral antibiotics	78	2.9%	7.30%	96.1%	29.9%	10.8%	57.1%	59.4%	81.1%
Parenteral anticonvulsants	67	4.6%	0.0%	69.3%	69.3%	2.9%	56.3%	9.2%	46.5%
Parenteral uterotonics	78	19.7%	0.0%	68.8%	77.30	45.3%	34.4%	24.6%	35.2%
Manual removal of placenta	78	0.0%	0.0%	69.7%	93.5%	8.2%	51.6%	1.6%	13.0%
Removal of retained products (manual vacuum aspiration)	76	0.0%	0.0%	62.7%	84.6%	5.5%	45.3%	1.6%	8.6%
Removal of retained products (dilation & curettage)	71	0.0%	0.0%	70.3%	50.0%	4.2%	53.1%	1.6%	7.5%
Assisted vaginal delivery (vacuum extraction)	78	0.0%	0.0%	69.3%	74.4%	5.6%	55.6%	1.6%	7.4%
Assisted vaginal delivery (forceps or vacuum)	71	0.0%	0.0%	61.3%	71.1%	5.6%	51.6%	0.0%	7.2%
Newborn resuscitation with bag and mask	71	0.0%	0.0%	54.1%	23.3%	1.4%	43.5%	4.8%	6.0%
**CEmONC functions**									
Blood transfusion	58	27.0%	12.5%	75.3%	89.7%	62.7%	32.8%	43.8%	20.3%
Caesarean surgery	62	17.9%	10.0%	37.8%	33.3%	45.1%	12.9%	27.7%	17.6%

* Detailed findings related to provision of signal functions are published elsewhere [13]*.*

Between 23% and 90% of facilities reported midwife involvement in CEmONC provision, depending on the signal function. Involvement of midwives was most often reported in manual removal of placenta, removal of retained products of conception, assisted vaginal delivery, administration of anticonvulsants and blood transfusions. Involvement of nurses was most often reported in blood transfusions (63% of facilities), as well as administration of uterotonics and cesarean deliveries (45% of facilities); only up to 11% of facilities reported involvement of nurses in other signal functions.

**Figure 2:**
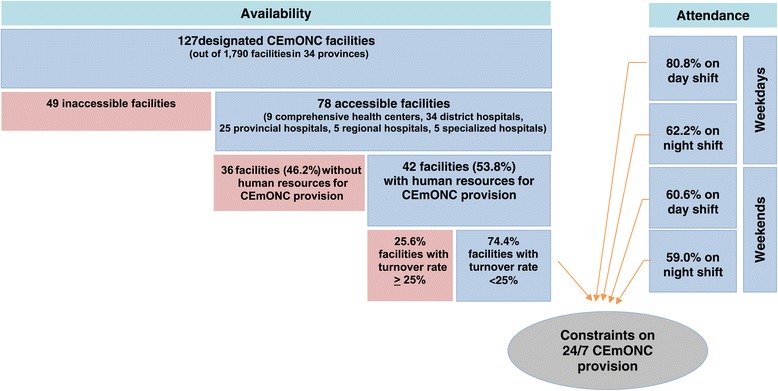
**Human resource challenges to CEmONC provision in Afghanistan.**

An illustration of the compounded effects of staff shortages, turnover, and gaps in actual duty hours on availability of CEmONC services is shown in Figure 2.

### Distribution of human resources to provide CEmONC services

As shown in Table 5, the presence of staff reported to provide all CEmONC functions differed significantly across facility types, but not geographic location (region) or location in urban versus rural areas. Only one-third of comprehensive health centers reported having staff who provide all nine CEmONC functions, compared with 44% of district hospitals, 64% of provincial hospitals, 80% of regional hospitals, and 80% of specialized hospitals *(p = 0.012)*.

**Table 5:** **Percentages of facilities meeting BPHS/EPHS staffing requirements and reporting provision ofCEmONC functions, by facility type, location, and region**

	**Number of facilities**	**Anesthetist**	**Lab technician**	**Medical doctor**	**Midwife**	**Nurse**	**Obstetrician/gynecologist**	**Pediatrician**	**Pharmacist**	**General surgeon**	**Staff who provide all BEmONC functions**	**Staff who provide all CEmONC functions**
**Health facility type**												
Comprehensive Health Center	9	55.6	77.8	77.8	88.9	66.7	11.1	N/A	N/A	33.3	55.6	33.3
District Hospital	34	91.2	70.6	79.4	70.6	0.0	5.9	26.5	14.7	20.6	61.8	44.1
Provincial Hospital	25	80.0	40.0	32.0	16.0	4.0	8.0	40.0	44.0	24.0	72.0	64.0
Regional Hospital	5	80.0	80.0	60.0	60.0	0.0	40.0	100.0	20.0	100.0	80.0	80.0
Specialized Hospital	5	100.0	100.0	60.0	80.0	0.0	80.0	40.0	60.0	40.0	100.0	80.0
*p-value*		*0.248*	*0.948*	*0.031*	*0.05*	*<0.001*	*<0.001*	*0.753*	*0.002*	*0.087*	*0.048*	*0.012*
**Location**												
0% urban	25	76.0	64.0	68.0	28.0	16.0	48.0	4.0	20.0	68.0	64.0	48.0
≤50% urban	36	83.3	52.8	58.3	22.2	33.3	33.3	5.6	5.6	38.9	66.7	55.6
>50% urban	17	94.1	88.2	58.8	47.1	23.5	64.7	47.1	0.0	70.6	76.5	58.8
*p-value*		*0.129*	*0.184*	*0.512*	*0.856*	*0.019*	*<0.001*	*0.424*	*0.471*	*0.257*	*0.423*	*0.477*
**Region**												
Central	24	83.3	62.5	50.0	58.3	0.0	20.8	45.8	25.0	25.0	62.5	54.2
East/Southeast	11	72.7	90.9	36.4	45.5	9.1	9.1	54.5	63.6	36.4	72.7	63.6
Northeast	11	100.0	72.7	81.8	63.6	9.1	9.1	63.6	18.2	36.4	72.7	72.7
West	7	85.7	42.9	57.1	57.1	0.0	14.3	42.9	14.3	28.6	14.3	14.3
North	19	78.9	52.6	84.2	63.2	21.1	10.5	31.6	15.8	26.3	89.5	52.6
Southwest	6	83.3	66.7	50.0	16.7	16.7	16.7	33.3	16.7	33.3	66.7	50.0
*p-value*		*0.955*	*0.319*	*0.057*	*0.594*	*0.031*	*0.518*	*0.263*	*0.145*	*0.91*	*0.395*	*0.514*

The percentage of facilities meeting the minimum staffing requirements set in BPHS and EPHS guidelines varied across facility types and well as location for some cadres. Gaps in obstetrician/gynecologist staffing were widespread across comprehensive health centers, district hospitals, and provincial hospitals with no more than 11% of these facilities meeting minimum staffing requirements for that cadre. Shortages of midwives and medical doctors were most substantial at provincial hospitals, with only 16% and 32% of facilities at that level meeting midwife and doctor staffing requirements, respectively. Finally, although two-thirds of comprehensive health centers met minimum staffing requirements for nurses, there were extreme shortages at all other facility levels with only 4% of provincial hospitals and no district, regional, or specialized hospitals meeting nurse staffing requirements. The percentage of facilities meeting staffing requirements for nurses also varied significantly by location, with the lowest proportions occurring in rural areas and central and western regions.

## Discussion

Along with access to skilled attendance at birth, universal access to EmONCis considered essential to reduce maternal mortality and this requires that all women and newborns with complications have rapid access to well-functioning facilities [17]. Each designated CEmONC facility is expected to serve as a referral site for several lower level facilities. Underperformance can therefore have substantial consequences for the broader population. Results of this study identify specific gaps between the availability and distribution of human resources for CEmONC and national policy requirements; these results can inform future policy and program considerations.

### Gaps in availability and distribution of human resources to provide CEmONC

While it was encouraging that all facilities had at least one midwife, and nearly all had at least one medical doctor and anesthetist, there are still severe gaps between the actual number of skilled providers and the recommended staffing paradigm within the BPHS and EPHS. These gaps may be the largest barrier Afghanistan faces with regard to increasing the availability of human resources for CEmONC, which is underscored by findings of a recent systematic review that, in developing countries, the most common supply-side barrier to maternal care is staff shortages [4]. Lack of obstetrician/gynecologists at more than half of accessible CEmONC facilities is of great concerning and likely contributes to an unmet need for cesarean section. This is the most serious gap and more strategic efforts to increase numbers of obstetricians/gynecologists and surgeons who can perform cesarean sections are required both short term and longer term, especially with marked population growth in Afghanistan. Additionally, absence of medical doctors and anesthetists at some facilities also affects the ability of facilities to provide surgical services.

Human resource shortages are further complicated by the absence of key personnel at night and on the weekends. CEmONC services should be available 24 hours a day, seven days a week, and assessment findings show that obstetrician/gynecologists, anesthetists, and surgeons contribute little to services during weekends and nightshifts. For example, only 31.5% of facilities with obstetrician/gynecologists on staff (15.3% of all facilities assessed) reported having the services of obstetrician/gynecologists during night shifts, leaving 84.7% of health facilities with no surgical capacity at night, which could result in significant delays in receiving appropriate care. Nominal availability of certain staff categories on the payroll of a health facility is not enough. In all areas of the country, essential staff must be available to treat women with obstetric complications upon arrival at the facility.

Staff working as a team to provide CEmONC services in the face of human resource shortages should be commended; however, reported involvement of some staff in provision of specific signal functions and apparent blurring of roles and responsibilities across cadres raises concerns. For example, some facilities reported involvement of pediatricians and laboratory technicians in cesarean surgeries. It is possible that this is a reporting aberration, as these cadres are not expected to have any involvement in cesarean surgeries. However, another explanation could be that the cadres involved undertake tasks beyond their intended scope of practice in emergency situations. While some may have gained on-the-job experience, others may be acting as needs arise without appropriate training, capacity, or authorization, which raises questions of effectiveness and accountability for care. In contrast, no facilities reported involvement of anesthetists in neonatal resuscitation, despite the fact that female doctors with anesthetist training are considered skilled birth attendants who should have the capacity to perform this function. To ensure women and their newborns have access to quality, lifesaving care, it is essential that all facilities are equipped with teams of staff that have complementary skill sets so that each cadre can maintain the roles and responsibilities they are trained for and authorized to provide.

Results from the distribution of human resources, such as the midwife staffing shortages at provincial hospitals, suggest that there are gaps in deployment planning for hospital midwives. Two types of pre-service midwifery education programs exist in Afghanistan: institutes of health sciences, which admit 12^th^ grade graduates, and community midwifery education schools, which admit 9^th^ grade graduates nominated by community leaders and selected by a local stakeholder committee after successful completion of entrance exams. Community midwifery education graduates are deployed to comprehensive health centers and district hospitals because the schools aim to deploy graduates to local health facilities, while institutes of health sciences graduates have no pre-determined deployment arrangements [18]. Although both programs require their graduates to achieve the core competencies recommended by the International Confederation of Midwives and both are licensed by the General Directorate of Human Resources of the Ministry of Public Health, the difference in deployment strategies between the two education programs and opportunities available to institutes of health sciences graduates to work in regional and specialized hospitals may lead to a gap in deployment of midwives to provincial hospitals.

### Recommendations

The Ministry of Public Health of Afghanistan recognizes that increased investment in both the number and the capacity of human resources for health is a national priority in order to strengthen their impact on population health [19,20]. Based on the identified gaps, we recommend specific areas that can be considered by the Ministry of Public Health for policy and program planning. First, pre-service education or in-service training should be provided to all female medical doctors in the seven signal functions that constitute BEmONC and to all female surgeons in all nine CEmONC signal functions. A more rational distribution of tasks and responsibilities across cadres is seen as a promising strategy for improving and increasing access and cost effectiveness, especially in remote facilities [21]. Overall surgical capacity in Afghanistan is inadequate, so strengthening CEmONC would also support surgical capacity to manage trauma and other emergencies [22].

Second, scaling up educational programs to produce more doctors, nurses, midwives, and other health professionals is urgent and essential, and should be supported by improved collaboration between the health and education sectors and the health systems that employ graduates [23].

Third, task shifting of additional CEmONC responsibilities to midwives should be considered as a strategy for minimizing the consequences of human resource supply shortages [21]. The assessment findings show that midwives are available in most facilities, have a relatively low turnover rate, and are available at night and during weekends at higher rates when compared to other categories. However, a recent evaluation revealed that facility protocols or management culture may prevent midwives from realizing their full scope of practice defined by the International Confederation of Midwives and endorsed by the Afghanistan Ministry of Public Health, including prescription of medications required to address some obstetric and neonatal complications, such as puerperal sepsis, unless a doctor is present [9]. To ensure that women with obstetric complications have timely access to care in facilities with essential staff vacancies or absentee doctors, midwives (and nurses with appropriate training) could be granted authority to prescribe drugs necessary for the signal functions of CEmONC. Shifting obstetric surgery, anesthesia, and abortion tasks to midwives may also be worthy of consideration, as this has been shown to be an effective strategy for optimizing health worker roles in other settings [24]. However, attention must first be given to ensuring all Afghan midwives are able to provide the full range of services outlined in their job descriptions, including administration of lifesaving medicines. Task shifting/sharing of specialist CEmONC responsibilities will require a series of advocacy efforts to allow a pilot test to train midwives to perform cesarean sections, changing legislation and protocols, mitigating potential resistance from professional groups, and—in the longer term—ensuring that midwives with more advance skills are recognized and compensated accordingly.

Fourth, human resource management policies and strategies must directly address imbalances in the distribution of the health workforce. Data on health workforce dynamics (including workforce growth, loss, and mobility) as well as gaps in services are vital to informing recruitment and workforce planning decisions [25]. If increasing access to CEmONC is a priority, this should be reflected in the design and regulation of systems for human resource management in the health sector, including how health workers are assigned to jobs, how transfers of staff between positions and locations are governed, and how services are organized to encourage productivity and retention of competent staff.

Finally, motivational factors—such as perceived underpayment, difficult living conditions, and lack of opportunities and education for family members—discourage some health workers, such as obstetrician/gynecologists, from accepting positions at lower level hospitals or facilities in more remote or insecure settings [9,26]. Given the human resource shortages and everyday challenges, staff may feel that they are not sufficiently compensated for the work that they do. In addition to increasing base salaries or hardship allowances, supporting spouses to find appropriate jobs through establishing linkages and collaboration with the Ministry of Labor and Social Affairs and other organizations, providing recommendation letters for spouses of health workers to facilitate their job search, and improving the housing and schooling facilities in and around health facilities could help encourage health workers to consider employment in areas with persistent staff shortages.

Certain limitations must be taken into account when considering the results and recommendations of this study. First, the data for this study was collected in late 2009 to early 2010, and there may have been changes in the availability and distribution of human resources for CEmONC services since that time. Although it is possible that the availability and distribution of some cadres or capacity of some facilities to provide CEmONC services may have improved, it may have also deteriorated due to increasing insecurity in many parts of the country. Second, data from facility record reviews and staff interviews may be subject to biases associated with each method. However, assessment tools were designed to draw on multiple sources of information and assessment team members trained to cross-check responses to ensure that findings presented are as accurate as the research methods allow.

This study remains the first effort to assess gaps in the availability and distribution of human resources for CEmONC provision in Afghanistan, and illustrates availability, distribution, and motivational challenges of health workforce scale-up in a setting where conflict destroyed much of the country’s health and health education infrastructure. A follow-up assessment or monitoring of current human resource for health availability and distribution is recommended to refine recommendations to address current policy and health system dynamics.

## Conclusions

Afghanistan faces several challenges in the availability and distribution of human resources for CEmONC service provision [27]. Increasing the number of staff who provide CEmONC services, through training of female doctors, midwives, nurses, and other cadres as well as task shifting, should be considered. Fundamental reforms are needed to increase the numbers and distribution of the necessary cadres across different facility levels and locationsto ensure all women and newborns in need of essential lifesaving services have access to timely appropriate care.

## Endnotes

^a^Obstetrician/gynecologist positions vacated in 2009 = 1; average number of obstetrician/gynecologists employed in 2009 = 2; obstetrician/gynecologist turnover rate = 1/2 = 50%.

^b^Obstetrician/gynecologist positions vacated in 2009 = 1; average number of obstetrician/gynecologists employed in 2009 = 0.5 (1 on staff in January 2009, 0 on staff in December 2009); obstetrician/gynecologist turnover rate = 1/0.5 = 200%.

## Abbreviations

BEmONC: Basic emergency obstetric and newborn care
BPHS: Basic package of health services
CEmONC: Comprehensive emergency obstetric and newborn care
EmONC: Emergency obstetric and newborn care
EPHS: Essential package of hospital services
